# Screening for Psychological Distress, Disaster-Related Experiences, and Newly Developed Mental Disorders among Residents Affected by the Great East Japan Earthquake: Implications for Suicide Prevention

**DOI:** 10.31662/jmaj.2025-0257

**Published:** 2025-11-28

**Authors:** Masatsugu Orui, Mana Kogure, Yuka Kotozaki, Taku Obara, Mami Ishikuro, Aoi Noda, Genki Shinoda, Keiko Murakami, Hirohito Metoki, Masahiro Kikuya, Yoshitake Takebayashi, Masaharu Maeda, Naoki Nakaya, Kozo Tanno, Atsushi Hozawa, The Tohoku Medical Megabank Project Study Group, Shinichi Kuriyama

**Affiliations:** 1 International Research Institute of Disaster Science, Tohoku University, Sendai, Japan; 2 Tohoku Medical Megabank Organization, Tohoku University, Sendai, Japan; 3 Graduate School of Medicine, Tohoku University, Sendai, Japan; 4 Iwate Medical Megabank Organization, Iwate Medical University, Yahaba, Iwate, Japan; 5 Tohoku University Hospital, Tohoku University, Sendai, Japan; 6 Graduate School of Medicine, The University of Tokyo, Tokyo, Japan; 7 Division of Public Health, Hygiene and Epidemiology, Tohoku Medical and Pharmaceutical University, Sendai, Japan; 8 Department of Hygiene and Public Health, Teikyo University School of Medicine, Tokyo, Japan; 9 Department of Health Risk Communication, School of Medicine, Fukushima Medical University, Fukushima, Japan; 10 Department of Disaster Psychiatry, School of Medicine, Fukushima Medical University, Fukushima, Japan

**Keywords:** suicide, psychological distress, binge drinking, disaster experiences, screening

## Abstract

**Introduction:**

This study aimed to evaluate the accuracy of screening using the six-item Kessler Psychological Distress Scale (K6) for suicide death after the Great East Japan Earthquake (GEJE) in combination with binge drinking, diagnosed history of mental disorders, disaster-related experiences, disaster stress symptoms, sleep status, and social network, which were used in the practical settings of disaster-related mental health services.

**Methods:**

This prospective cohort study spanned the period from 2013 to 2021. Among the Tohoku Medical Megabank Project sample, 27,335 affected residents and 61,157 residents living within the disaster-stricken area (i.e., those who experienced partial or no house damage and did not evacuate even if they lived in the disaster-stricken area) of the GEJE were analyzed using receiver operating characteristic curve (ROC) analyses of the K6 score in combination with the following related factors: binge drinking, disaster experiences (loss of a family member, relatives and friends, and decrease in income), disaster-related stress symptoms (recollection of disaster experiences, physical reactions due to recalling the disaster), diagnosed history of mental disorders (depression and post-traumatic stress disorder [PTSD]), sleep status, and social network.

**Results:**

In the ROC analyses, when the K6 score was combined with relevant variables, newly developed PTSD after the GEJE (area under the curve [AUC]: 0.878 [95% confidence interval (CI): 0.773-0.982]), disaster-related stress symptom of recollection of disaster experiences (AUC: 0.849 [95% CI: 0.714-0.985]), and decreased income (AUC: 0.835 [95% CI: 0.726-0.945] yielded higher AUCs than did the K6 score alone (AUC: 0.681 [95% CI: 0.567-0.794]).

**Conclusions:**

When screening using K6 for suicide death, combining the K6 score with newly developed PTSD, recollection-related disaster stress symptoms, and decreased income could generate more accurate predictions of suicide after the disaster. We hope these findings will contribute to minimizing post-disaster suicide in the context of disaster-related mental health services. (297 words)

## Introduction

Devastating natural disasters and their aftermaths are known to cause psychological distress in the individuals affected, which can lead to suicidality ^(1), (2)^. The 2011 Great East Japan Earthquake (GEJE) undoubtedly had a severe impact on mental health among those affected ^(3), (4), (5), (6), (7), (8), (9)^. Against this backdrop, our earlier research revealed that the GEJE was followed by an increase in suicide rates in the disaster-stricken area ^(10), (11), (12)^. After the GEJE, disaster-related mental health services, such as psychological first aid, screening for individuals requiring observation or support, and counseling services, were offered early in the post-disaster period to address the broad mental health impacts among affected residents, thereby helping to prevent suicide ^(13), (14), (15), (16), (17)^.

In disaster mental health activities after the GEJE, the six-item Kessler Psychological Distress Scale (K6) ^(18), (19)^―a widely used screening checklist for identifying individuals at high risk―was used extensively in practical settings (e.g., K6 score ≥13 is defined as an individual at high risk) ^(20), (21)^. Mental health screening has been conducted using K6 in accordance with Japan’s disaster-related mental health guidelines ^(22), (23)^, which is also used to screen for suicidality ^(24)^.

A previous study found both severe and moderate psychological distress to be associated with an increased risk of suicide death among the Japanese general population during a non-disaster phase ^(25)^. This finding indicates that a certain number of suicide cases occur even among those whose K6 scores are lower than 13 points ^(25)^; therefore, relying solely on K6 screening may not be ideal for suicide prevention after disasters. Thus, other checklists that evaluate related factors, such as binge drinking, disaster-related experiences, traumatic stress, and history of mental disorders, are also used as important screening tools in disaster-related mental health services in Japan ^(20), (21)^.

However, simply evaluating K6 scores including other related factors―binge drinking, disaster-related experiences, traumatic stress, and history of mental disorders―is not sufficient among residents affected by disasters to determine whether these screening efforts can prevent suicide death after disasters. To our knowledge, no studies have yet been reported that examine the accuracy of suicide death screening using the K6 score among residents affected by disasters. Therefore, to generate solid evidence for suicide prevention after disasters, we aimed to evaluate the accuracy of screening for suicide death after the GEJE using the K6―alongside assessing for binge drinking, disaster-related experiences, traumatic stress, and newly developed mental disorders―in a large-scale cohort study established after the GEJE. We hypothesized that (1) screening for suicide prevention through K6 alone would not be sufficiently accurate and that (2) screening using K6 in conjunction with assessments of binge drinking, disaster experiences, disaster-related stress symptoms, and newly developed mental disorders would be more accurate than using K6 alone.

## Methods

### Disaster-related mental health services after the GEJE

According to the Japanese Fire and Disaster Management Agency, the GEJE disaster led to 15,844 deaths, with more than 90% of the deaths attributed to drowning due to a massive tsunami ^(26)^.

After the GEJE, disaster-related mental health services were conducted from an early stage across multiple settings―such as shelters, temporary housing, and post-disaster public housing―in the disaster-stricken area, which have been primarily organized by the prefectural government. These services aimed to support affected residents, alleviate psychological distress, and prevent suicide. Screening to identify individuals at high risk of mental health problems plays a crucial role in these disaster-related services. When residents are recognized as being in a high-risk mental health state, public health nurses or other health professionals reach out to provide counseling and other forms of psychological support; if necessary, the residents are referred to specialized institutions. These disaster-related mental health services are modeled on suicide prevention activities that have been performed in the non-disaster phase for communities in Niigata and Akita prefectures, which have been evaluated for the effectiveness of suicide prevention ^(27), (28)^.

### Study design and linkage of death data of vital statistics

This is a prospective cohort study. The Tohoku Medical Megabank Project (TMM) began in 2013 with the aim of assisting medical and health services in overcoming the damage from the GEJE by supporting survivors and implementing personalized health care. The TMM encompasses two prospective cohort studies in Miyagi and Iwate prefectures. One is a population-based adult cohort study―the TMM Community-Based Cohort Study (TMM CommCohort Study)―in which more than 80,000 residents aged 20 to 74 years were recruited from 2013 to 2016. The other cohort study is a birth and three-generation cohort study―the TMM Birth and Three-Generation Cohort Study (TMM BirThree Cohort Study)―which recruited more than 70,000 residents from 2013 to 2017 by first enrolling pregnant women at obstetric clinics or hospitals, along with their children, partners, parents, and extended family members.

**Figure 1.**
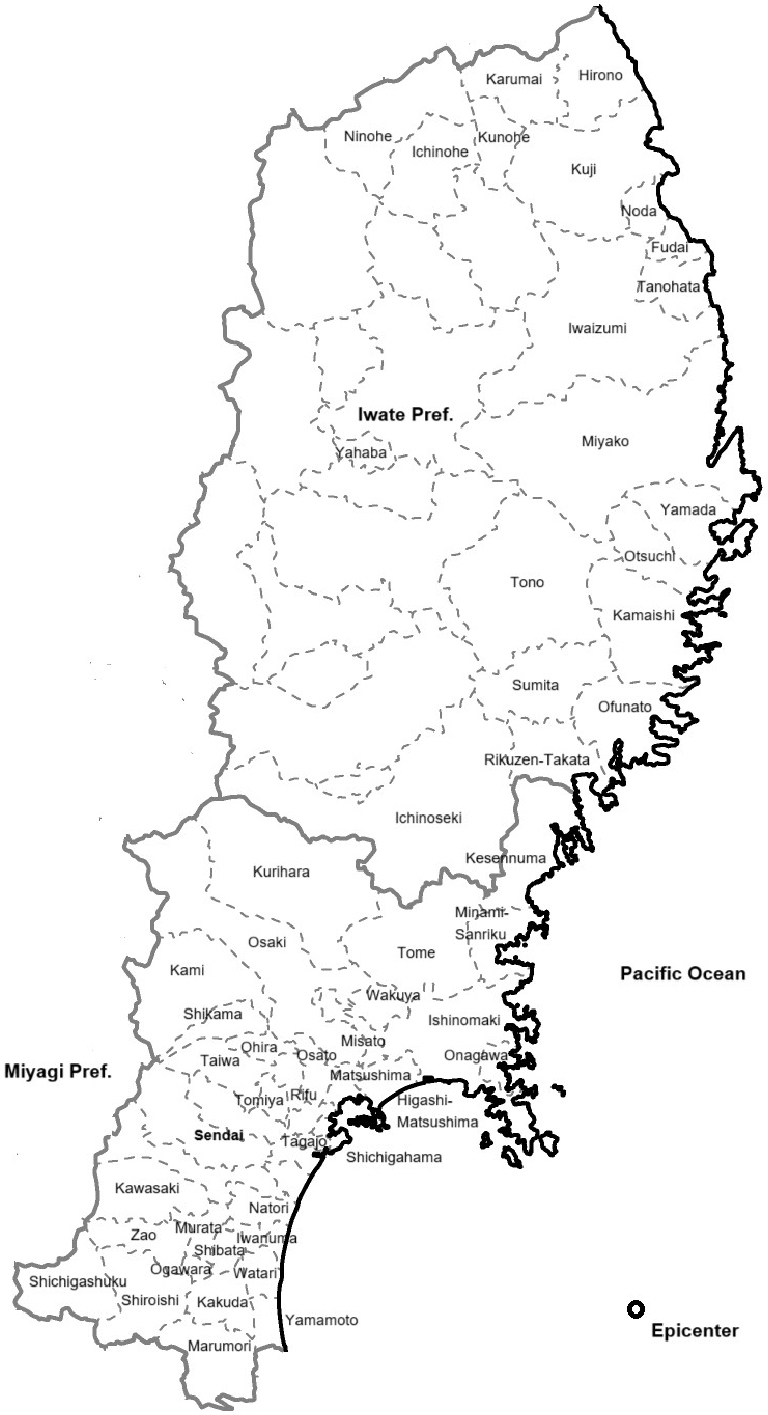
Municipalities in Iwate Prefecture and Miyagi Prefecture.

The regions targeted by these two cohorts are shown in Figure 1. They include all 35 municipalities in Miyagi Prefecture and 20 municipalities (Miyako City, Ofunato City, Kuji City, Tono City, Ichinoseki City, Rikuzen-Takata City, Kamaishi City, Ninohe City, Yahaba town, Sumita town, Otsuchi town, Yamada town, Iwaizumi town, Tanohata village, Fudai village, Karumai town, Noda village, Kunohe village, Hirono town, and Ichinohe town) in Iwate Prefecture ^(29)^.

To link death data from Japan’s Ministry of Health, Labour, and Welfare, we gathered extensive vital statistical data for the period of 2013 through 2021, which encompassed data regarding age, sex, address, dates of birth and death, and cause of death. Subsequently, we matched cohort participants on the basis of these variables to determine causes of death.

### Outcome

In this study, the outcome set suicide death. We defined suicide death among the cohort participants using the International Classification of Diseases, 10th Revision, codes X60-84.

### Study period

This study period was set from 2013 to 2021. Given participants in these cohort studies were registered in 2013 or later, this study targeted the mid- to long-term and recovery phases after the GEJE. It should be noted that the participants’ lifestyles, social networks, socioeconomic statuses, and mental health statuses did not reflect the immediate aftermath of the GEJE.

### Participants

**Figure 2.**
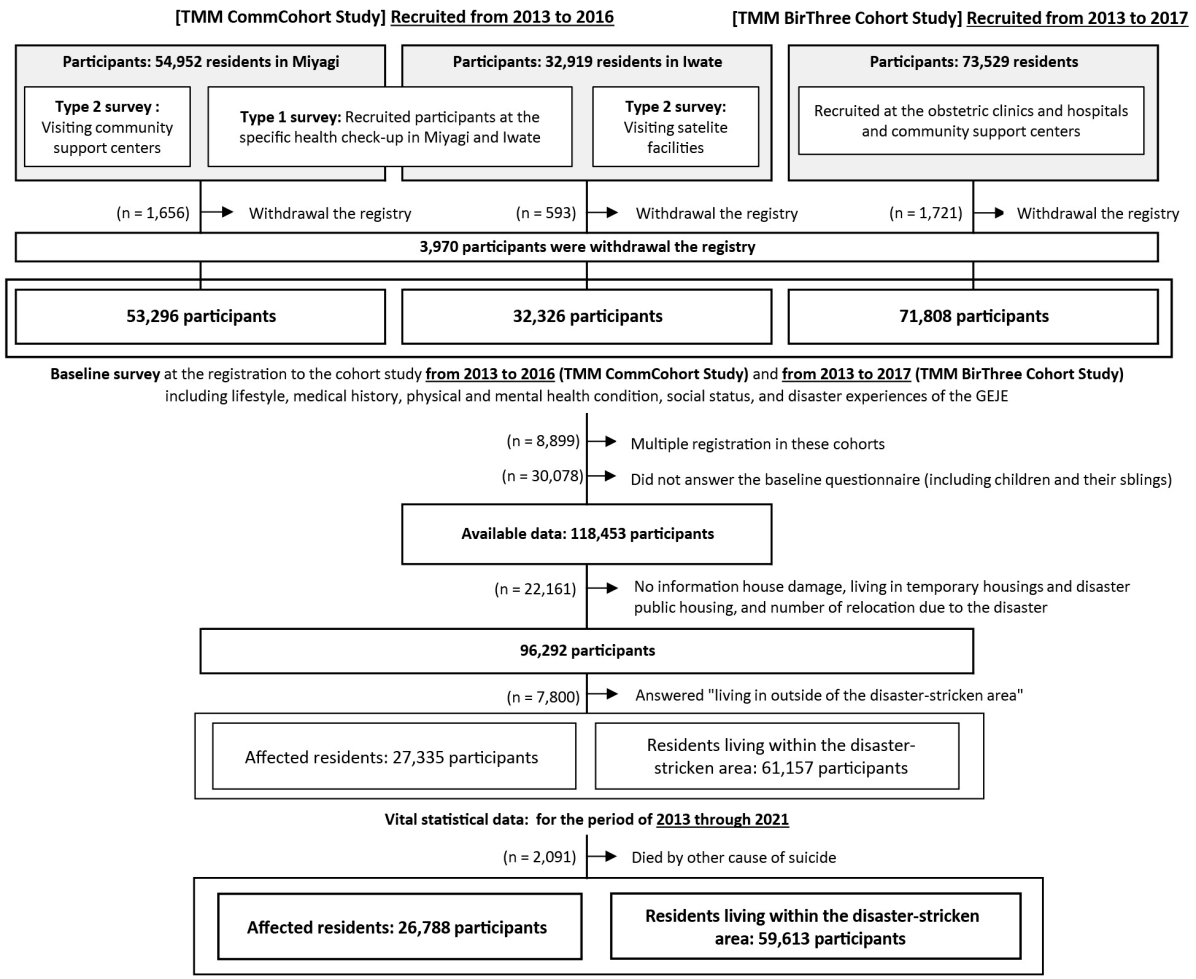
Participants in the TMM CommCohort Study and BirThree Cohort Study and subjects analyzed. TMM: Tohoku Medical Megabank Project.

The participants in the two cohort studies are presented in Figure 2. The TMM CommCohort Study enrolled 54,952 participants in Miyagi, and 32,326 participants in Iwate. The TMM BirThree Cohort Study enrolled 73,529 participants. Among participants in the TMM CommCohort study and the TMM BirThree Cohort study, 3,970 withdrew their registration as of December 2023. A total of 8,899 participants were enrolled in both cohorts, and 30,078 participants did not complete the baseline questionnaire. As a result, 118,453 participants had baseline questionnaire data available for analysis. We excluded 22,161 participants owing to missing information on (1) house damage caused by the GEJE, (2) whether they lived in temporary or post-disaster public housing, and (3) the number of evacuations they experienced owing to the GEJE, and excluded those who answered, “living outside of the disaster-stricken area.”

In this study, we defined “affected residents” as participants who had experienced house damage (totally destroyed, mostly destroyed, or half destroyed), lived in temporary or post-disaster public housing, and evacuated to shelters at least once (n = 27,335). Those who experienced partial or no house damage and no evacuations, even if they lived in the disaster-stricken area, were classified as “residents living within the disaster-stricken area” (n = 61,157).

### Measurements

At the baseline survey, we collected the following demographic and disaster-related variables: sex (men, women), age group (18-39, 40-64, and ≥65 years), educational attainment (junior/high school, vocational school/junior college, and university/graduate school), marital status (married or unmarried/divorced/bereaved), housing damage due to the GEJE (totally destroyed, mostly destroyed, half destroyed, partially destroyed, not destroyed, or did not live in the disaster-stricken area), living conditions (whether residing in temporary or post-disaster public housing), and the number of evacuations to shelters or temporary housing.

Besides, the following variables collected at the baseline survey―psychological distress, binge drinking, disaster experiences, disaster-related stress symptoms, diagnosed mental history of disorders, sleep status, and social network―were selected as related variables because they were used commonly in practical settings in disaster-related mental health activities ^(16), (20)^. Furthermore, these variables have been identified as risk factors for suicide ideation in previous studies on disaster-related mental health ^(30), (31), (32), (33), (34), (35)^.

### Psychological distress (K6)

We used the K6 scale to assess current psychological distress. The K6 scale screens for non-specific serious mental illnesses, including Diagnostic and Statistical Manual of Mental Disorders, Fourth Edition mood and anxiety disorders, indicating psychological distress within the past 30 days. Scores on the K6 range from 0 to 24 points. A score of 0-4 points indicates probable absence of psychological distress; 5-12 points indicates probable mild-to-moderate distress, and 13 points or more indicates serious psychological distress ^(18)^. This study used the Japanese version of the K6 scale, which has been empirically validated as an independent screening tool for mental distress ^(19)^.

### Binge drinking

Participants were asked the following questions about their drinking habits: “Do you drink alcohol more than once a month?” and “If you are a drinker, what kind of alcohol and how much do you drink on a typical drinking day?” We then defined “binge drinkers” based on the frequency of drinking and the amount of alcohol consumed on a typical drinking day. “Binge drinking” was defined as drinking alcohol every day and consuming more than six drinks per day (≥66 g of ethanol per day). “Six drinks” was defined as 180 mL of spirits (e.g., whiskey or brandy), 600 mL of wine, 1,500 mL of beer, or 540 mL of Japanese sake ^(36), (37)^.

### Disaster experiences and disaster-related stress symptoms

We assessed participants’ disaster experiences by asking whether any family, relatives, or friends died or went missing because of the GEJE. To evaluate changes in income after the GEJE, we asked participants whether they had changed their income, offering three response options: “increased income,” “decreased income,” or “no change.”

We also assessed disaster-related stress symptoms using investigator-designed queries: “Do you recall or dream about the disaster, even if you would prefer not to?” (recollection of disaster experiences) and “Do you have any physical reactions when you think about the disasters (e.g., heart palpitations, shortness of breath, sweating, dizziness)?” (physical reactions due to recalling the disaster).

### History of diagnosed mental disorders

Besides the question about disaster-related stress symptoms, the history of diagnosed mental disorders was collected through self-reported answers. Respondents were asked: “Have medical doctors diagnosed you with any of the following diseases before/after the GEJE?” We classified any diagnosed cases into “developed pre-disaster” and “newly developed after the GEJE.” We focused on depression and post-traumatic stress disorder (PTSD), given previous studies have reported that these disorders are more likely to develop after devastating natural disasters ^(38), (39)^.

### Sleep status

Sleep status was measured using the Athens Insomnia Scale (AIS), a validated screening tool for sleep status ^(40)^. This study used the Japanese version of the AIS, which includes the following items: sleep induction, awakenings during the night, final awakening earlier than desired, total sleep duration, overall quality of sleep, sense of well-being during the day, daytime physical and mental functioning, and daytime sleepiness. Participants who scored 6 or higher were classified as having sleep problems ^(41)^.

### Social networks

Social networks were evaluated using the Lubben Social Network Scale-6 (LSNS-6), Japanese version ^(42)^. The scale comprises six questions: (1) “How many relatives do you see or hear from at least once a month?”; (2) “How many relatives do you feel comfortable talking with about private matters?”; (3) “How many relatives do you feel close to such that you could call on them for help?”; (4) “How many of your friends do you see or hear from at least once a month?”; (5) “How many friends do you feel comfortable talking with about private matters?”; and (6) “How many friends do you feel close to such that you could call on them for help?” Participants responded on a five-point scale (0 = none, 1 = one, 2 = two, 3 = three or four, 4 = five to eight, 5 = nine or more). Respondents with 11 points or fewer were classified as socially isolated ^(43)^.

### Data analysis

We excluded 2,091 participants who had died from causes other than suicide death. First, we performed a simple tabulation, then conducted univariate logistic regression analysis to evaluate the association with suicide death after the GEJE, adjusted for cohort group, sex, age group, educational attainment, and marital status.

Furthermore, receiver operating characteristic (ROC) curve analyses were performed to assess the predictive ability of the K6 score in combination with single variables in discriminating suicide death, as follows: (1) K6 score alone, and K6 score with (2) binge drinking, (3) social network, (4) sleep problems, (5) diagnosed mental disorders after the GEJE, (6) disaster-related experiences, and (7) disaster stress symptoms. We then compared the area under the ROC curve (AUC) for the K6 score combined with each variable and without each variable. AUC values higher than 0.80 are generally considered clinically useful, whereas values lower than 0.80 are considered of limited clinical utility ^(44)^.

Statistical significance was evaluated using two-sided, design-based tests at a 5% significance level, with analyses conducted using Stata 18 (StataCorp, College Station, TX, USA).

### Ethical considerations

Our study protocol for the TMM CommCohort Study and the TMM BirThree Cohort Study was approved by Ethical Research Committee at Tohoku University Graduate School of Medicine (2013-4-103, approval date: May 10, 2013; latest revised 2023-4-040, approval date: June 21, 2023). Moreover, this study protocol has conformed to the provisions of the Declaration of Helsinki. Informed consent has been obtained from all participants of TMM CommCohort Study and TMM BirThree Cohort Study.

## Results

### Basic participant characteristics

The participants’ basic characteristics are listed in Table 1. Among the affected residents, the proportions of individuals with newly developed depression and PTSD, disaster-related experiences, and disaster-related stress symptoms were higher. Moreover, the proportions of moderate (K6 score: 5-12) and severe (K6 score: 13 or more) psychological distress were high. Sleep disturbances were also slightly more prevalent among the affected residents. Incidentally, the crude suicide mortality rate per 100,000 participants in each group―affected residents and residents within the disaster-stricken area―were 10.8 and 7.6, respectively.

**Table 1.** Basic Characteristic of Participants in TMM CommCohort Study and BirThree Cohort Study.

		Affected residents		Residents living within the disaster-stricken area	
		(n=27,335)	(％)	(n=61,157)	(％)
Sex	men	11,932	(43.7)	23,962	(39.2)
	women	15,403	(56.3)	37,195	(60.8)
Age group	18-39	6,998	(25.6)	6,232	(10.2)
	40-64	11,943	(43.7)	30,392	(49.7)
	≥65 years	8,394	(30.7)	24,533	(40.1)
Educational attainment	junior/high school	16,245	(60.1)	39,838	(66.0)
	vocational school/junior college	6,563	(24.3)	13,878	(23.0)
	university/graduate school	4,243	(15.7)	6,622	(11.0)
Marital status	married	21,365	(79.0)	49,101	(81.2)
	unmarried/divorced/bereaved	5,691	(21.0)	11,366	(18.8)
Psychological distress (K6 score)	0-4 points	14,429	(53.0)	37,157	(61.1)
	5-12 points	10,602	(38.9)	20,328	(33.4)
	≥13 points	2,189	(8.0)	3,348	(5.5)
Binge drinking	more than 6 drinks/day and every day	1,115	(4.1)	2,294	(3.8)
Social networks (LSNS-6)	≤11 points	6,799	(25.8)	14,553	(24.8)
Sleep problems (AIS)	≥6 points	7,035	(25.8)	13,138	(21.5)
History of diagnosed mental disorders	depression developed pre-disaster	645	(2.4)	1,330	(2.2)
	depression newly developed after the GEJE	310	(1.1)	312	(0.5)
	PTSD developed pre-disaster	49	(0.2)	81	(0.1)
	PTSD newly developed after the GEJE	224	(0.8)	125	(0.2)
Disaster experiences	loss of a family member	1,525	(5.8)	916	(1.6)
	loss of relatives	7,580	(28.8)	10,231	(17.5)
	loss of friends	5,830	(22.1)	7,219	(12.3)
Changes in income after the GEJE	increased in income/no change	11,190	(67.0)	23,997	(72.4)
	decreased income	5,504	(33.0)	9,141	(27.6)
Disaster-related stress symptoms	recollection of disaster experiences	3,721	(14.0)	3,963	(6.7)
	physical reactions due to recalling the disaster	1,659	(6.2)	1,842	(3.1)
Number of suicide cases	Total number (2013 through 2021)		26		41
	/100,000 participants (annualized)		10.8		7.6

AIS: Athens Insomnia Scale; GEJE: Great East Japan Earthquake; LSNS-6: Lubben Social Network Scale-6; PTSD: post-traumatic stress disorder; TMM: Tohoku Medical Megabank Project.

### Association with suicide death

Among the affected residents, depression newly developed after the GEJE, PTSD developed pre-disaster, and PTSD newly developed after the GEJE were a higher proportion in suicide cases than those who lived within the disaster-stricken area (Table 2).

**Table 2.** Basic Characteristics of Suicide Cases in Affected Residents and Residents Living within the Disaster-Stricken Area.

		Affected residents				Residents living within the disaster-stricken area			
		Suicide death (+)		Suicide death (-)		Suicide death (+)		Suicide death (-)	
		(n=26)	(%)	(n=26,762)	(%)	(n=41)	(%)	(n=59,572)	(%)
Sex	men	19	(0.16)	11,577	(99.84)	25	(0.11)	22,925	(99.89)
	women	7	(0.05)	15,185	(99.95)	16	(0.04)	36,647	(99.96)
Age group	18-39	5	(0.07)	6,985	(99.93)	7	(0.11)	6,213	(99.89)
	40-64	13	(0.11)	11,782	(99.89)	22	(0.07)	29,938	(99.93)
	≥65 years	8	(0.10)	7,995	(99.90)	12	(0.05)	23,421	(99.95)
Educational attainment	junior/high school	12	(0.08)	15,835	(99.92)	27	(0.07)	38,667	(99.93)
	vocational school/junior college	5	(0.08)	6,473	(99.92)	6	(0.04)	13,661	(99.96)
	university/graduate school	9	(0.21)	4,180	(99.79)	8	(0.12)	6,456	(99.88)
Marital status	married	19	(0.09)	20,951	(99.91)	29	(0.06)	47,869	(99.94)
	unmarried/divorced/bereaved	6	(0.11)	5,544	(99.89)	12	(0.11)	11,045	(99.89)
Psychological distress (K6)	0-4 points	7	(0.05)	14,129	(99.95)	15	(0.04)	36,156	(99.96)
	5-12 points	13	(0.13)	10,380	(99.87)	16	(0.08)	19,828	(99.92)
	≥13 points	6	(0.28)	2,144	(99.72)	10	(0.30)	3,275	(99.70)
Binge drinking	more than 6 drinks/day and every day	1	(0.09)	1,080	(99.91)	4	(0.18)	2,209	(99.82)
Social networks (LSNS-6)	≤11 points	11	(0.17)	6,643	(99.83)	17	(0.12)	14,139	(99.88)
Sleep problems (AIS)	≥6 points	13	(0.19)	6,880	(99.81)	17	(0.13)	12,802	(99.87)
History of diagnosed mental disorders	depression developed pre-disaster	5	(0.80)	622	(99.20)	8	(0.62)	1,286	(99.38)
	depression newly developed after the GEJE	2	(0.66)	301	(99.34)	1	(0.33)	306	(99.67)
	PTSD developed pre-disaster	1	(2.08)	47	(97.92)	0	(0.0)	80	(100.0)
	PTSD newly developed after the GEJE	3	(1.40)	211	(98.60)	0	(0.0)	119	(100.0)
Disaster experiences	loss of a family member	1	(0.07)	1,493	(99.93)	1	(0.11)	886	(99.89)
	loss of relatives	8	(0.11)	7,380	(99.89)	10	(0.10)	9,939	(99.90)
	loss of friends	8	(0.14)	5,655	(99.86)	5	(0.07)	6,997	(99.93)
Changes in income after the GEJE	decreased income	7	(0.13)	5,398	(99.87)	9	(0.10)	8,926	(99.90)
Disaster-related stress symptoms	recollection of disaster experiences	5	(0.14)	3,611	(99.86)	5	(0.13)	3,827	(99.87)
	physical reactions due to recalling the disaster	5	(0.31)	1,609	(99.69)	2	(0.11)	1,802	(99.89)

AIS: Athens Insomnia Scale; GEJE: Great East Japan Earthquake; K6: six-item Kessler Psychological Distress Scale; LSNS-6: Lubben Social Network Scale-6; PTSD: post-traumatic stress disorder.

In univariate logistic regression analysis, individuals with reduced social networks (LSNS-6 ≤11), moderate and severe psychological distress (K6 5-12 and K6 ≥13), sleep problems (AIS ≥6), or a diagnosis of depression or PTSD (pre-disaster or newly developed) were significantly associated with suicide death among affected residents. In addition, disaster-related stress symptoms, such as physical reactions when recalling the disaster, were specific risk factors (Table 3). Incidentally, we found little evidence of a notable inverse relationship between men and women, and each age group (Supplementary Table 1 and 2).

**Table 3.** Univariate Logistic Analysis of Risk for Suicide Death among Participants.

		Affected residents (n=26,788)			Residents living within the disaster-stricken area (n=59,613)		
		OR*	(95% CI)	p-value	OR*	(95% CI)	p-Value
Psychological distress (K6)	5-12 points	2.688	(1.051-6.877)	0.039	2.042	(1.002-4.160)	0.049
	≥13 points	7.718	(2.226-23.59)	<0.001	7.552	(3.268-17.45)	<0.001
Binge drinking	more than 6 drinks/day and every day	0.680	(0.090-5.139)	0.709	1.912	(0.663-5.514)	0.231
Social networks (LSNS-6)	≤11 points	2.144	(0.945-4.867)	0.068	2.086	(1.096-3.969)	0.025
Sleep problems (AIS)	≥6 points	3.630	(1.643-8.020)	0.001	3.030	(1.597-5.750)	0.001
History of diagnosed mental disorders	depression developed pre-disaster	7.986	(2.667-23.92)	<0.001	10.38	(4.710-22.86)	<0.001
	depression newly developed after the GEJE	9.906	(2.272-43.19)	0.002	5.036	(0.683-37.15)	0.113
	PTSD developed pre-disaster	28.50	(3.557-228.4)	0.002	-	-	-
	PTSD newly developed after the GEJE	20.96	(6.050-72.62)	<0.001	-	-	-
Disaster experiences	loss of a family member	0.736	(0.097-5.581)	0.767	1.781	(0.243-13.03)	0.570
	loss of relatives	1.640	(0.665-4.042)	0.283	1.963	(0.947-4.068)	0.070
	loss of friends	1.700	(0.678-4.262)	0.258	1.131	(0.440-2.907)	0.798
Changes in income after the GEJE	decreased income	1.277	(0.431-3.781)	0.659	2.191	(0.906-5.301)	0.082
Disaster-related stress symptoms	recollection of disaster experiences	2.178	(0.789-6.012)	0.133	2.364	(0.917-6.091)	0.075
	physical reactions due to recalling the disaster	6.043	(2.217-16.86)	0.001	1.937	(0.462-8.113)	0.366

OR: odds ratio; CI: confidence interval; *OR: Adjusted by cohort type, sex, age group, educational attainment, and marital status. AIS: Athens Insomnia Scale; GEJE: Great East Japan Earthquake; K6: six-item Kessler Psychological Distress Scale; LSNS-6: Lubben Social Network Scale-6; PTSD: post-traumatic stress disorder.

### Predicting suicide death in ROC analyses

The AUC of the K6 score alone was 0.681 (95% confidence interval [CI]: 0.567-0.794) for affected residents. When the K6 score was combined with additional variables, newly developed PTSD after the GEJE (AUC: 0.878 [95% CI: 0.773-0.982]), the disaster stress symptom of recollection of disaster experiences (AUC: 0.849 [95% CI: 0.714-0.985]), and decreased income (AUC: 0.835 [95% CI: 0.726-0.945] were significantly higher than that of the AUC without each variable, which yielded much higher AUC values combined with these variables than did K6 alone (Table 4).

**Table 4.** ROC Curve Analyses Were Performed to Assess the Predictive Ability of the K6 Score in Combination with Other Related Variables.

		Affected residents (n = 26,788)			Residents living within the disaster-stricken area (n = 59,613)		
		AUC	(95% CI)	p-Value	AUC	(95% CI)	p-Value
K6 score alone		0.681	(0.567-0.794)	-	0.699	(0.602-0.882)	-
K6 with binge drinking	more than 6 drinks/day and every day	-	-	-	0.728	(0.394-1.000)	0.833
K6 with social networks	LSNS-6 ≤11 points	0.627	(0.447-0.805)	0.457	0.613	(0.168-0.7308)	0.107
K6 with sleep problems	AIS ≥6 points	0.702	(0.555-0.849)	0.319	0.758	(0.644-0.873)	0.117
K6 with diagnosed mental disorders	depression developed pre-disaster	0.720	(0.474-0.966)	0.566	0.695	(0.517-0.874)	0.696
	depression newly developed after the GEJE	0.773	(0.377-1.000)	0.509	-	-	-
	PTSD developed pre-disaster	-	-	-	-	-	-
	PTSD newly developed after the GEJE	0.878	(0.773-0.982)	0.004	-	-	-
K6 with disaster experiences	loss of a family member	-	-	-	-	-	-
	loss of relatives	0.746	(0.601-0.890)	0.245	0.692	(0.506-0.877)	0.997
	loss of friends	0.721	(0.519-0.922)	0.468	0.836	(0.675-0.997)	0.078
K6 with changes in income after the GEJE	decreased income	0.835	(0.726-0.945)	0.014	0.699	(0.504-0.894)	0.729
K6 with disaster-related stress symptoms	recollection of disaster experiences	0.849	(0.714-0.985)	0.020	0.866	(0.779-0.952)	0.005
	physical reactions due to recalling the disaster	0.780	(0.589-0.970)	0.165	0.969	(0.958-0.981)	<0.001

AUC: area under the curve; CI: confidence interval; *p-Value: comparison with K6 and related variables vs K6 without related variables. AIS: Athens Insomnia Scale; GEJE: Great East Japan Earthquake; K6: six-item Kessler Psychological Distress Scale; LSNS-6: Lubben Social Network Scale-6; PTSD: post-traumatic stress disorder; ROC: receiver operating characteristic.

## Discussion

We identified in ROC curve analysis combining the K6 score with newly developed PTSD after the GEJE or with the disaster stress symptom of recollection of disaster experiences improved the accuracy of suicide prediction compared with using K6 alone. We consider the utility of K6, a commonly used measure in Japanese disaster settings, for suicide prevention screening. According to our ROC analysis, the AUC for K6 alone was 0.681 (95% CI: 0.567-0.794), suggesting that K6 alone is not sufficiently accurate for predicting suicide death. In contrast, combining the K6 score with newly developed PTSD, with disaster stress symptoms (e.g., physical reactions to recalling the disaster), and with decreased income significantly increased predictive accuracy.

It goes without saying that PTSD and traumatic stress are well-established risk factors for suicide ^(45), (46)^. Even with a self-reported measure of diagnosed PTSD, the K6-plus-newly developed PTSD approach still yielded better predictive accuracy than did K6 alone. Moreover, in comparing disaster experiences and associated symptoms, the latter (e.g., physical reactions to disaster recollections) showed greater predictive power for suicide death than did the experience of losing family or friends. Although losing a loved one is undoubtedly stressful, our findings emphasize the importance of screening for stress responses rather than simply cataloging disaster-related events. Residents with disaster-related stress symptoms were supported by disaster-related mental health services, which have provided continuous counseling through outreach to help reduce these residents’ psychological burden. In fact, frequent outreach efforts may have contributed to relief in traumatic stress symptoms, leading to the successful conclusion of the support activities ^(16)^. For this backdrop, it would be essential to screen for K6 in addition to disaster-related stress symptoms in the setting of disaster-related mental health services, and to provide continuous support to those affected residents.

We also found that combining the K6 score with decreased income due to the GEJE improved suicide prediction accuracy among affected residents, consistent with previous studies linking income reduction after a devastating disaster to severe psychological distress or post-traumatic stress ^(47), (48)^.

Overall, these findings indicate that relying solely on the K6 score is insufficient for suicide prevention in post-disaster contexts. Instead, screening protocols should incorporate additional factors―especially disaster-related stress symptoms, newly developed PTSD, and changes in income―to more effectively identify individuals at high risk. Incidentally, the positive predictive value of K6 ≥13 points with newly developed PTSD was 3.85% among the affected residents, which was greater than that of K6 ≥5 points with newly developed PTSD (1.65%). Thus, in the context of disaster-related mental health activities, a K6 score of greater than 13 points would be appropriate (Supplementary Table 3).

Regarding differences between affected residents and residents living within the disaster-stricken area, this study found that there was a difference in binge drinking and disaster experiences of loss of family members; binge drinking and experiencing the loss of a family member presented a lower risk of suicide death among affected residents, whereas both factors posed a higher risk among those living within the disaster-stricken area. The disparity in the level of disaster-related mental health services after the GEJE may have an impact on these results. Affected individuals with binge drinking behaviors and the experience of loss of a family member were primarily targeted for counseling in the disaster-related mental health services ^(20), (21)^, whereas residents living within the disaster-stricken area had limited access to disaster-related mental health interventions.

Several limitations merit consideration. First, the cause of suicide death was unclear; moreover, this study did not ascertain whether the participants actually received disaster-related mental health services. Second, our classification―affected residents and residents living within the disaster-stricken area―was based on self-reported data. Third, there are inherent representativeness issues, given cohort participants tend to be more interested in healthy behaviors ^(49)^, and our study sample may not fully reflect all affected residents in the disaster-stricken region. Fourth, all variables―including diagnosed mental disorders―were self-reported, raising the possibility of response bias. Fifth, because the TMM CommCohort and BirThree Cohort Studies began in 2013, our findings may not apply to the immediate post-disaster period because risk factors for suicide death could differ shortly after such an event. Fifth, the number of suicide cases was small, limiting the statistical power to detect certain risk factors or fully evaluate predictive accuracy. Furthermore, the relative variables among suicide cases were exceedingly low (e.g, binge drinking and loss of family were just two cases, respectively). As a result, combining K6 exposure with multiple factors was challenging to evaluate. Finally, this study area is limited to specific region in Japan.

Despite these limitations, this large-scale cohort study uniquely integrates several disaster-specific questionnaire items with official cause-of-death data. To our knowledge, this was the first study to investigate suicide risk and predictive accuracy for suicide death in a cohort established after the GEJE, offering valuable insights into post-disaster suicide prevention measures.

In conclusion, our study identified, combining the K6 score with newly developed PTSD, the disaster stress symptom of recollection of disaster experiences, and changes in income improved suicide prediction accuracy compared with using K6 alone when the ROC curve analyses. We hope these findings will help minimize post-disaster suicide death by guiding practical disaster-related mental health services in the future. (3,417 words)

### Data availability statement

According to regulations from the Ministry of Health, Labour and Welfare, permission is required to use vital statistical data; therefore, it must be requested in advance to the corresponding author. All data except vital statistical data used to support the findings may be released on application to the Tohoku Medical Megabank Organization.

## Article Information

### Acknowledgments

Our research members are as follows: https://www.megabank.tohoku.ac.jp/english/a240901/

This study was permitted officially for the use of data of vital statistics from the Ministry of Health, Labour on the basis of Article 33 of the Statistics Act.

### Author Contributions

Masatsugu Orui conceptualized and designed this study, conducted the initial analyses, drafted the initial manuscript, and revised the manuscript.

Shinichi Kuriyama conceptualized, designed, and organized the TMM CommCohort Study and BirThree Cohort Study, and reviewed the manuscript.

Atsushi Hozawa, Kozo Tanno, Naoki Nakaya, Mana Kogure, and Yuka Kotozaki managed the implementation of the TMM CommCohort Study and reviewed the manuscript.

Masahiro Kikuya, Hirohito Metoki, Taku Obara, Mami Ishikuro, Aoi Noda, Genki Shinoda, and Keiko Murakami managed the implementation of the TMM BirThree Cohort Study and reviewed the manuscript.

Masatsugu Orui, Mana Kogure, and Yuka Kotozaki performed the linkage of vital statistical data and the information of baseline questionnaires.

Masaharu Maeda and Yoshitake Takebayashi conceptualized this study and critically reviewed the manuscript.

All authors approved the final manuscript as submitted and agreed to be accountable for all aspects of the work.

### Conflicts of Interest

None

### Approval code issued by the institutional review board and the name of the institution(s)

This study was approved by the Ethical Research Committee at Tohoku University Graduate School of Medicine (TMM CommCohort Study: 2012-4-617, approval date: March 21, 2013; TMM BirThree Cohort Study: 2013-4-103, approval date: May 10, 2013). Informed consent was obtained from all participants in the TMM CommCohort Study and TMM BirThree Cohort Study.

## Supplement

Supplementary Material

## References

[1] Murphy SA. Status of natural disaster victims’ health and recovery 1 and 3 years later. Res Nurs Health. 1986;9(4):331-40. 3643610 10.1002/nur.4770090410

[2] Kiliç C, Ulusoy M. Psychological effects of the November 1999 earthquake in Turkey: an epidemiological study. Acta Psychiatr Scand. 2003;108(3):232-8. 12890279 10.1034/j.1600-0447.2003.00119.x

[3] Sone T, Nakaya N, Sugawara Y, et al. Longitudinal association between time-varying social isolation and psychological distress after the Great East Japan Earthquake. Soc Sci Med. 2016;152:96-101. 26851408 10.1016/j.socscimed.2016.01.037

[4] Kunii Y, Suzuki Y, Shiga T, et al. Severe psychological distress of evacuees in evacuation zone caused by the Fukushima daiichi nuclear power plant accident: the Fukushima health management survey. PLoS One. 2016;11(7):e0158821. 27391446 10.1371/journal.pone.0158821 PMC4938533

[5] Oe M, Maeda M, Nagai M, et al. Predictors of severe psychological distress trajectory after nuclear disaster: evidence from the Fukushima Health Management Survey. BMJ Open. 2016;6(10):e013400. 10.1136/bmjopen-2016-013400 PMC5073554 27798033

[6] Tanji F, Tomata Y, Sekiguchi T, et al. Period of residence in prefabricated temporary housing and psychological distress after the Great East Japan Earthquake: a longitudinal study. BMJ Open. 2018;8(5):e018211. 10.1136/bmjopen-2017-018211 PMC5942419 29730612

[7] Orui M, Nakayama C, Moriyama N, et al. Those who have continuing radiation anxiety show high psychological distress in cases of high post-traumatic stress: the Fukushima nuclear disaster. Int J Environ Res Public Health. 2021;18(22):12048. 34831804 10.3390/ijerph182212048 PMC8623122

[8] Kunii Y, Usukura H, Utsumi Y, et al. Review of mental health consequences of the great East Japan earthquake through long-term epidemiological studies: the Shichigahama health promotion project. Tohoku J Exp Med. 2022;257(2):85-95. 35569933 10.1620/tjem.2022.J039

[9] Tsubota-Utsugi M, Yonekura Y, Suzuki R, et al. Psychological distress in responders and nonresponders in a 5-year follow-up health survey: the RIAS study. J Epidemiol. 2022;32(12):527-34. 33840653 10.2188/jea.JE20200617 PMC9643786

[10] Orui M, Sato Y, Tazaki K, et al. Delayed increase in male suicide rates in tsunami disaster-stricken areas following the great east japan earthquake: a three-year follow-up study in Miyagi Prefecture. Tohoku J Exp Med. 2015;235(3):215-22. 25765170 10.1620/tjem.235.215

[11] Orui M, Suzuki Y, Maeda M, et al. Suicide rates in evacuation areas after the Fukushima daiichi nuclear disaster. Crisis. 2018;39(5):353-63. 29618266 10.1027/0227-5910/a000509 PMC6263751

[12] Takebayashi Y, Hoshino H, Kunii Y, et al. Characteristics of disaster-related suicide in Fukushima Prefecture after the nuclear accident. Crisis. 2020;41(6):475-82. 32141328 10.1027/0227-5910/a000679 PMC8208296

[13] Kim Y. Great East Japan earthquake and early mental-health-care response. Psychiatry Clin Neurosci. 2011;65(6):539-48. 22003986 10.1111/j.1440-1819.2011.02270.x

[14] Suzuki Y, Kim Y. The great east Japan earthquake in 2011; toward sustainable mental health care system. Epidemiol Psychiatr Sci. 2012;21(1):7-11. 22670406 10.1017/s2045796011000795

[15] Fukasawa M, Suzuki Y, Nakajima S, et al. Systematic consensus building on disaster mental health services after the great East Japan earthquake by phase. Disaster Med Public Health Prep. 2015;9(4):359-66. 25905559 10.1017/dmp.2015.13

[16] Orui M, Harada S, Hayashi M, et al. Practical report on long-term disaster mental health services following the great East Japan earthquake: psychological and social background of evacuees in Sendai City in the mid- to long-term post-disaster period. Disaster Med Public Health Prep. 2017;11(4):439-50. 28327208 10.1017/dmp.2016.157

[17] Takahashi S, Takagi Y, Fukuo Y, et al. Acute mental health needs duration during major disasters: A phenomenological experience of disaster psychiatric assistance teams (DPATs) in Japan. Int J Environ Res Public Health. 2020;17(5):1530. 32120917 10.3390/ijerph17051530 PMC7084937

[18] Kessler RC, Barker PR, Colpe LJ, et al. Screening for serious mental illness in the general population. Arch Gen Psychiatry. 2003;60(2):184-9. 12578436 10.1001/archpsyc.60.2.184

[19] Furukawa TA, Kawakami N, Saitoh M, et al. The performance of the Japanese version of the K6 and K10 in the World Mental Health Survey Japan. Int J Methods Psychiatr Res. 2008;17(3):152-8. 18763695 10.1002/mpr.257 PMC6878390

[20] Orui M, Saeki S, Harada S, et al. Practical report of disaster-related mental health interventions following the great East Japan earthquake during the COVID-19 pandemic: potential for suicide prevention. Int J Environ Res Public Health. 2021;18(19):10424. 34639724 10.3390/ijerph181910424 PMC8507691

[21] Maeda M, Harigane M, Horikoshi N, et al. Long-term, community-based approach for affected people having problems with mental health and lifestyle issues after the 2011 Fukushima disaster: the Fukushima health management survey. J Epidemiol. 2022;32(suppl XII):S47-56. 36464300 10.2188/jea.JE20210178 PMC9703932

[22] Guidelines for local mental health care activities after a disaster [Internet]. Mental Health Research Center, National Center of Neurology and Psychiatry Japan. 2024 [cited 2024 Nov 11]. Available from: https://saigai-kokoro.ncnp.go.jp/contents/pdf/mental_info_guide_en.pdf

[23] Tachibana A, Kitamura H, Shindo M, et al. Psychological distress in an earthquake-devastated area with pre-existing high rate of suicide. Psychiatry Res. 2014;219(2):336-40. 24928758 10.1016/j.psychres.2014.01.028

[24] Sakurai K, Nishi A, Kondo K, et al. Screening performance of K6/K10 and other screening instruments for mood and anxiety disorders in Japan. Psychiatry Clin Neurosci. 2011;65(5):434-41. 21851452 10.1111/j.1440-1819.2011.02236.x

[25] Tanji F, Tomata Y, Zhang S, et al. Psychological distress and completed suicide in Japan: a comparison of the impact of moderate and severe psychological distress. Prev Med. 2018;116:99-103. 30219687 10.1016/j.ypmed.2018.09.007

[26] IRI. DeS, Tohoku University. Statistical database on the great East Japan earthquake [Internet]. Global Centre for Disaster Statistics. 2015 [cited 2025 Mar 4]. Available from: http://www.geje-gcds.jp/en/#search-result2

[27] Takahashi K, Naito H, Morita M, et al. Suicide prevention for the elderly in Matsunoyama Town, Higashikubiki County, Niigata Prefecture: psychiatric care for elderly depression in the community. Seishin Shinkeigaku Zasshi. 1998;100(7):469-85. Japanese. 9778997

[28] Motohashi Y, Kaneko Y, Sasaki H. Community-based suicide prevention program in Japan using a health promotion approach. Environ Health Prev Med. 2004;9(1):3-8. 21432331 10.1265/ehpm.9.3 PMC2723381

[29] Kuriyama S, Yaegashi N, Nagami F, et al. The Tohoku medical Megabank project: design and mission. J Epidemiol. 2016;26(9):493-511. 27374138 10.2188/jea.JE20150268 PMC5008970

[30] Kessler RC, Galea S, Jones RT, et al. Mental illness and suicidality after Hurricane Katrina. Bull World Health Organ. 2006;84(12):930-9. 17242828 10.2471/blt.06.033019 PMC1852424

[31] Tang W, Xu D, Li B, et al. The relationship between the frequency of suicidal ideation and sleep disturbance factors among adolescent earthquake victims in China. Gen Hosp Psychiatry. 2018;55:90-7. 30448743 10.1016/j.genhosppsych.2018.09.013

[32] Jafari H, Heidari M, Heidari S, et al. Risk factors for suicidal behaviours after natural disasters: A systematic review. Malays J Med Sci. 2020;27(3):20-33. 32684803 10.21315/mjms2020.27.3.3 PMC7337952

[33] Chen XY, Zhou Y, Shi X, et al. Longitudinal associations between adolescents’ trajectory membership of depressive symptoms and suicidality in young adulthood: a 10-year cohort of Chinese Wenchuan earthquake survivors. Epidemiol Psychiatr Sci. 2020;29:e175. 33070799 10.1017/S2045796020000827 PMC7681160

[34] Gibson R, Whealin JM, Dasaro CR, et al. Prevalence and correlates of suicidal ideation in World Trade Center responders: results from a population-based health monitoring cohort. J Affect Disord. 2022;306:62-70. 35283182 10.1016/j.jad.2022.03.011 PMC12713793

[35] Fischer IC, Nichter B, Feldman DB, et al. Purpose in life protects against the development of suicidal thoughts and behaviors in U.S. veterans without a history of suicidality: a 10-year, nationally representative, longitudinal study. J Affect Disord. 2023;340:551-4. 37557988 10.1016/j.jad.2023.08.040

[36] Health Japan 21 (the second term) [Internet]. National Institute of Health and Nutrition. 2024 [cited 2025 Mar 4]. Available from: https://www.nibiohn.go.jp/eiken/kenkounippon21/en/

[37] Yokoyama T. National Health Promotion Measures in Japan: health Japan 21(the second term). J Natl Inst Public Health. 2020;69(1):14-24.

[38] Sakuma A, Takahashi Y, Ueda I, et al. Post-traumatic stress disorder and depression prevalence and associated risk factors among local disaster relief and reconstruction workers fourteen months after the Great East Japan Earthquake: a cross-sectional study. BMC Psychiatry. 2015;15:58. 25879546 10.1186/s12888-015-0440-y PMC4374405

[39] Ando S, Kuwabara H, Araki T, et al. Mental health problems in a community after the great East Japan earthquake in 2011: A systematic review. Harv Rev Psychiatry. 2017;25(1):15-28. 28059933 10.1097/HRP.0000000000000124

[40] Soldatos CR, Dikeos DG, Paparrigopoulos TJ. The diagnostic validity of the Athens Insomnia Scale. J Psychosom Res. 2003;55(3):263-7. 12932801 10.1016/s0022-3999(02)00604-9

[41] Okajima I, Nakajima S, Kobayashi M, et al. Development and validation of the Japanese version of the Athens Insomnia Scale. Psychiatry Clin Neurosci. 2013;67(6):420-5. 23910517 10.1111/pcn.12073

[42] Lubben J, Blozik E, Gillmann G, et al. Performance of an abbreviated version of the Lubben Social Network Scale among three European Community-dwelling older adult populations. Gerontologist. 2006;46(4):503-13. 16921004 10.1093/geront/46.4.503

[43] Kurimoto A, Awata S, Ohkubo T, et al. Reliability and validity of the Japanese version of the abbreviated Lubben Social Network Scale. Nihon Ronen Igakkai Zasshi. 2011;48(2):149-57. In Japanese. 21778631 10.3143/geriatrics.48.149

[44] Çorbacıoglu SK, Aksel G. Receiver operating characteristic curve analysis in diagnostic accuracy studies: a guide to interpreting the area under the curve value. Turk J Emerg Med. 2023;23(4):195-8. 38024184 10.4103/tjem.tjem_182_23 PMC10664195

[45] Fox V, Dalman C, Dal H, et al. Suicide risk in people with post-traumatic stress disorder: a cohort study of 3.1 million people in Sweden. J Affect Disord. 2021;279:609-16. 33190111 10.1016/j.jad.2020.10.009 PMC7758737

[46] Forehand JA, Dufort V, Gradus JL, et al. Association between post-traumatic stress disorder severity and death by suicide in US military veterans: retrospective cohort study. Br J Psychiatry. 2022:1-7. 10.1192/bjp.2022.110 PMC9947187 35997207

[47] Hossain A, Ahmed B, Rahman T, et al. Household food insecurity, income loss, and symptoms of psychological distress among adults following the Cyclone Amphan in coastal Bangladesh. PLoS One. 2021;16(11):e0259098. 34727102 10.1371/journal.pone.0259098 PMC8562802

[48] Shiga T, Zhang W, Ohira T, et al. Socioeconomic status, damage-related conditions, and PTSD following the Fukushima-daiichi nuclear power plant accident: the Fukushima Health Management Survey. Fukushima J Med Sci. 2021;67(2):71-82. 34456222 10.5387/fms.2020-24 PMC8460284

[49] Harlow BL, MacLehose RF, Smolenski DJ, et al. Disparate rates of new-onset depression during the menopausal transition in 2 community-based populations: real, or really wrong? Am J Epidemiol. 2013;177(10):1148-56. 23589585 10.1093/aje/kws365 PMC3649637

