# Supplementary material for: Screening for Psychological Distress, Disaster-Related Experiences, and Newly Developed Mental Disorders among Residents Affected by the Great East Japan Earthquake: Implications for Suicide Prevention
Source: JMA J. 2025 Nov 28;9(1):77–88. doi: 10.31662/jmaj.2025-0257 (PMC12889190; doi:10.31662/jmaj.2025-0257)
Supplement: Supplementary Material [file 2433-3298-9-1-0077-s001.pdf]

Supplementary Table 1 Univariate logistic analysis of risk for suicide death among affected residents (sex)

		Men (n=11,596)			Women (n=15,192)		
		OR*	(95%CI)	p-value	OR*	(95%CI)	p-value
Psychological distress (K6)	5–12 points	2.984	(0.992-8.982)	0.052	1.792	(0.298-10.78)	0.524
	≥13 points	8.208	(2.116-31.84)	0.002	5.594	(0.776-40.32)	0.088
Binge drinking	more than 6-drink/day and everyday	0.697	(0.091-5.312)	0.728	-	-	-
Social networs (LSNS-6)	≤11 points	2.547	(0.956-6.786)	0.061	1.314	(0.251-6.870)	0.746
Sleep problems (AIS)	≥6 points	4.624	(1.813-11.79)	0.001	1.948	(0.434-8.754)	0.384
History of diagnosed mental disorders	depression developed pre-disaster	5.303	(1.170-24.04)	0.031	14.99	(2.826-79.51)	0.001
	depression newly developed after the GEJE	7.604	(0.970-59.63)	0.054	19.68	(2.169-178.6)	0.008
	PTSD developed pre-disaster	60.23	(6.489-559.1)	<0.001	-	-	-
	PTSD newly developed after the GEJE	23.74	(5.172-108.9)	<0.001	16.27	(1.879-140.9)	0.011
Disaster experiences	loss of a family member	-	-	-	3.123	(0.352-27.68)	0.306
	loss of relatives	1.305	(0.434-3.925)	0.636	2.273	(0.440-11.75)	0.327
	loss of friends	1.109	(0.348-3.533)	0.861	3.954	(0.771-20.29)	0.099
Changes in income after the GEJE	decreased income	1.552	(0.480-5.012)	0.463	-	-	-
Disaster-related stress symptoms	recollection of disaster experiences	1.847	(0.512-6.670)	0.349	2.478	(0.472-13.02)	0.283
	physical reactions due to recalling the disaster	6.711	(1.863-24.17)	0.004	4.635	(0.891-24.12)	0.068

* OR: Adjusted by cohort type, sex, age gropup, educational attainment and marital status

Supplementary Table 2 Univariate logistic analysis of risk for suicide death among affected residents (age groups)

		18-39 years (n=6,990)			40-64 years (n=11,795)			≥ 65 years (n=8,003)		
		OR*	(95%CI)	p-value	OR*	(95%CI)	p-value	OR*	(95%CI)	p-value
Psychological distress (K6)	5–12 points	0.313	(0.052-1.900)	0.206	1.912	(0.536-6.821)	0.318	2.034	(0.407-10.17)	0.387
	≥13 points	-	-	-	3.421	(0.612-19.13)	0.161	11.35	(1.802-71.45)	0.010
Binge drinking	more than 6-drink/day and everyday	-	-	-	1.210	(0.149-9.857)	0.858	-	-	-
Social networs (LSNS-6)	≤11 points	0.836	(0.093-7.495)	0.873	2.816	(0.870-9.113)	0.084	2.285	(0.500-10.45)	0.287
Sleep problems (AIS)	≥6 points	2.586	(0.429-15.61)	0.300	4.107	(1.287-13.10)	0.017	3.594	(0.886-14.58)	0.073
History of diagnosed mental disorders	depression developed pre-disaster	31.65	(5.119-195.7)	<0.001	2.681	(0.333-21.61)	0.354	9.097	(1.076-76.92)	0.043
	depression newly developed after the GEJE	-	-	-	18.69	(3.833-91.16)	<0.001	-	-	-
	PTSD developed pre-disaster	-	-	-	41.56	(4.670-369.9)	0.001	-	-	-
	PTSD newly developed after the GEJE	205.6	(15.80-2674.1)	<0.001	10.30	(1.275-83.27)	0.029	20.14	(2.358-171.9)	0.006
Disaster experiences	loss of a family member	-	-	-	1.136	(0.141-9.123)	0.904	-	-	-
	loss of relatives	2.314	(0.249-21.47)	0.460	1.767	(0.527-5.926)	0.357	1.004	(0.181-5.574)	0.997
	loss of friends	6.804	(1.113-41.58)	0.038	1.730	(0.499-6.000)	0.387	0.470	(0.055-4.051)	0.492
Changes in income after the GEJE	decreased income	3.139	(0.324-30.38)	0.323	0.856	(0.228-3.221)	0.819	1.153	(0.071-18.78)	0.920
Disaster-related stress symptoms	recollection of disaster experiences	13.31	(2.111-83.92)	0.006	1.507	(0.320-7.088)	0.604	0.885	(0.105-7.445)	0.911
	physical reactions due to recalling the disaster	12.085	(1.134-128.6)	0.039	6.142	(1.577-23.93)	0.009	3.451	(0.405-29.38)	0.257

* OR: Adjusted by cohort type, sex, age gropup, educational attainment and marital status

Supplementary Table 3 Positive predictable value by K6 ≥ 13 and ≥5 points

Affected residents

	with relative variables	suicide (+)	Total	(%), PPV	without relative variables	suicide (+)	Total	(%)
K6 ≥13 points	alone	6	2,150	0.28%	alone	6	2,150	0.28%
	binge drinking (+)	1	89	1.12%	binge drinking (-)	5	2,053	0.24%
	sleep problems (+)	5	1,446	0.35%	sleep problems (-)	1	700	0.14%
	LSNS-6 ≤11 points (+)	2	947	0.21%	LSNS-6 ≤11points (-)	4	1,127	0.35%
	pre-disaster depression (+)	3	178	1.69%	pre-disaster depression (-)	3	1,972	0.15%
	newly developed depression (+)	1	118	0.85%	newly developed depression (-)	3	1,955	0.15%
	pre-disaster PTSD (+)	0	17	0.00%	pre-disaster PTSD (-)	6	2,133	0.28%
	newly developed PTSD (+)	3	78	3.85%	newly developed PTSD (-)	3	2,066	0.15%
	loss of family member (+)	0	147	0.00%	loss of family member (-)	4	2,081	0.19%
	loss of relatives (+)	1	618	0.16%	loss of relatives (-)	3	1,463	0.21%
	loss of friends (+)	2	506	0.40%	loss of friends (-)	2	1,575	0.13%
	decreased income (+)	2	516	0.39%	decreased income (-)	1	809	0.12%
	recollection of disaster experiences (+)	3	688	0.44%	recollection of disaster experiences (-)	3	1,408	0.21%
	physical reaction due to recalling disaster (+)	3	445	0.67%	physical reaction due to recalling disaster (-)	3	1,642	0.18%
K6 ≥5 points	alone	19	12,543	0.15%	alone	19	12,543	0.15%
	binge drinking (+)	1	508	0.20%	binge drinking (-)	18	11,982	0.15%
	sleep problems (+)	12	5,183	0.23%	sleep problems (-)	9	7,330	0.12%
	LSNS-6 ≤11 points (+)	8	3,878	0.21%	LSNS-6 ≤11points (-)	11	8,217	0.13%
	pre-disaster depression (+)	5	474	1.05%	pre-disaster depression (-)	14	12,069	0.12%
	newly developed depression (+)	2	249	0.80%	newly developed depression (-)	15	12,148	0.12%
	pre-disaster PTSD (+)	1	42	2.38%	pre-disaster PTSD (-)	18	12,501	0.14%
	newly developed PTSD (+)	3	182	1.65%	newly developed PTSD (-)	16	12,530	0.13%
	loss of family member (+)	1	789	0.13%	loss of family member (-)	16	11,320	0.14%
	loss of relatives (+)	7	3,600	0.19%	loss of relatives (-)	10	8,509	0.12%
	loss of friends (+)	7	2,795	0.25%	loss of friends (-)	10	9,315	0.11%
	decreased income (+)	7	2,848	0.25%	decreased income (-)	5	5,002	0.10%
	recollection of disaster experiences (+)	5	2,600	0.19%	recollection of disaster experiences (-)	12	9,619	0.12%
	physical reaction due to recalling disaster (+)	5	1,293	0.39%	physical reaction due to recalling disaster (-)	12	10,885	0.11%

PPV: Positive Predictable Value

Residents living within the disaster-stricken area

		suicide (+)	Total	(%), PPV		suicide (+)	Total	(%)
K6 ≥13 points	alone	10	3,285	0.30%	alone	10	3,285	0.30%
	binge drinking (+)	2	144	1.39%	binge drinking (-)	8	3,118	0.26%
	sleep problems (+)	9	2,081	0.43%	sleep problems (-)	1	1,191	0.08%
	LSNS-6 ≤11 points (+)	4	1,557	0.26%	LSNS-6 ≤11points (-)	6	1,618	0.37%
	pre-disaster depression (+)	4	317	1.26%	pre-disaster depression (-)	6	2,968	0.20%
	newly developed depression (+)	0	99	0.00%	newly developed depression (-)	8	3,106	0.26%
	pre-disaster PTSD (+)	0	27	0.00%	pre-disaster PTSD (-)	10	3,285	0.30%
	newly developed PTSD (+)	0	40	0.00%	newly developed PTSD (-)	10	3,240	0.31%
	loss of family member (+)	0	58	0.00%	loss of family member (-)	9	3,083	0.29%
	loss of relatives (+)	3	506	0.59%	loss of relatives (-)	6	2,635	0.23%
	loss of friends (+)	2	427	0.47%	loss of friends (-)	8	2,714	0.29%
	decreased income (+)	3	631	0.48%	decreased income (-)	5	1,281	0.39%
	recollection of disaster experiences (+)	4	626	0.64%	recollection of disaster experiences (-)	6	2,540	0.24%
	physical reaction due to recalling disaster (+)	2	431	0.46%	physical reaction due to recalling disaster (-)	8	2,723	0.29%
K6 ≥5 points	alone	26	23,129	0.11%	alone	26	23,129	0.11%
	binge drinking (+)	3	882	0.34%	binge drinking (-)	23	22,124	0.10%
	sleep problems (+)	16	8,791	0.18%	sleep problems (-)	9	14,294	0.06%
	LSNS-6 ≤11 points (+)	11	7,106	0.15%	LSNS-6 ≤11points (-)	14	15,212	0.09%
	pre-disaster depression (+)	7	914	0.77%	pre-disaster depression (-)	19	22,215	0.09%
	newly developed depression (+)	0	230	0.00%	newly developed depression (-)	23	22,743	0.10%
	pre-disaster PTSD (+)	0	65	0.00%	pre-disaster PTSD (-)	26	23,064	0.11%
	newly developed PTSD (+)	0	100	0.00%	newly developed PTSD (-)	26	23,017	0.11%
	loss of family member (+)	0	382	0.00%	loss of family member (-)	24	21,778	0.11%
	loss of relatives (+)	7	4,039	0.17%	loss of relatives (-)	14	18,121	0.08%
	loss of friends (+)	4	2,942	0.14%	loss of friends (-)	20	19,218	0.10%
	decreased income (+)	6	3,994	0.15%	decreased income (-)	8	9,266	0.09%
	recollection of disaster experiences (+)	5	2,585	0.19%	recollection of disaster experiences (-)	19	19,800	0.10%
	physical reaction due to recalling disaster (+)	2	1,378	0.15%	physical reaction due to recalling disaster (-)	22	20,940	0.11%

PPV: Positive Predictable Value
